# Epidemic of Influenza at Camp Sherman, Ohio

**Published:** 1919

**Authors:** 


***The Epidemic of Influenza at Camp Sherman, Ohio.*** Major A. FRIEDLANDER, Major C. P. MCCORD, Captain F. G. SLADEN and Lieutenant G. W. WHEELER, M. C., U. S. Army. *Journal of the American Medical Association*, November 16, 1918.

In this epidemic, out of a population of 33,044 soldiers, 10,979 contracted the disease. Secondary to the influenza, there occurred a high incidence of pneumonia affecting 2,001 patients. The outstanding features of the epidemic were: two-thirds of the 1st group of cases occurred among men who had been inducted into service within the previous month; the incidence of the disease decreased with the length of residence in camp; the severity of the infection occasioned a high mortality; and all cases were attributable to acute inflammatory pulmonary pneumonia or edema.

*Bacteriology.* (1) Smears and cultures of sputum of influenza patients have exhibited the pneumococcus as the predominating organism.

(2) Cultures from swabbing of the throat and nasopharynx exhibited 54 o/o pneumococci and 4 o/o hemolytic streptococci.

(3) Cultures from sputum of patients after development of pneumonia exhibited pneumococci in 80 o/o of cases.

(4) Blood cultures from patients after development of pneumonia were sterile in 94 o/o of cases and exhibited pneumococci in 6 o/o.

(5) Postmortem cultures showed pneumococci in 53 o/o of cases and the streptococcus hemolyticus in 47 o/o.

The influenza bacilli has not been demonstrated as the causative organism.

*Clinical Manifestations.* The clinical manifestations necessitate a division into 4 types which represent a progression in severity.

Type 1, characterized by coryza and bronchitis.

Type 2, true influenza.

Type 3 comprised a group with subjective signs without demonstrable objective evidence of pneumonia.

Type 4, acute pulmonary inflammatory edema.

*Complications.* In the majority of cases with complications, pneumonia was the natural outcome. Other complications were slight : profuse epistaxis without nasal ulceration. Later acute catarrhal otitis media were frequent, but without danger.

*Therapy.* (1) *Influenza.* Undue exposure was avoided, and rest in bed, indoors, free purgation, gargles, acetysalicylic acid and Dover's powders constituted the general plan of treatment.

(2) *Acute Inflammatory Pulmonary Edema.* This condition must be regarded as the most fulminant type of respiratory influenza, against which no special measure was of avail.

(3) *Secondary Acute Bronchopneumonia.* Diet, fresh air, rest, mild purgations, etc., were recommended. All cases were digitalized and reliance placed on soluble caffein salt for quick stimulation. Strichnin in large doses hypodermically had a distinct value in the existing asthenia.

*General Measures.* The camp was quarantined. Theatres, post exchanges, Y. M. C. A. building, etc., were closed, barracks thoroughly cleaned, ventilation insisted upon, etc. A section of the camp was transformed into hospital wards and the base hospital reserved for cases of pneumonia.

